# Advances in Dynamization of Plate Fixation to Promote Natural Bone Healing

**DOI:** 10.3390/jcm13102905

**Published:** 2024-05-14

**Authors:** Michael Bottlang, Sunil S. Shetty, Connor Blankenau, Jennifer Wilk, Stanley Tsai, Daniel C. Fitzpatrick, Lawrence J. Marsh, Steven M. Madey

**Affiliations:** 1 Biomechanics Laboratory, Legacy Research Institute, Portland, OR 97232, USAcblankenau@biomechresearch.org (C.B.); stsai@biomechresearch.org (S.T.); madey@summitdocs.com (S.M.M.); 2 Department of Comparative Medicine, Legacy Research Institute, Portland, OR 97232, USA; jenwilk@lhs.org; 3 Slocum Center for Orthopedics and Sports Medicine, Eugene, OR 97401, USA; danfitz@mac.com; 4 Department of Orthopedics, University of Iowa Hospitals and Clinics, Iowa City, IA 52242, USA; j-marsh@uiowa.edu

**Keywords:** axial dynamization, fracture healing, callus, interfragmentary motion, plate, screw

## Abstract

The controlled dynamization of fractures can promote natural fracture healing by callus formation, while overly rigid fixation can suppress healing. The advent of locked plating technology enabled new strategies for the controlled dynamization of fractures, such as far cortical locking (FCL) screws or active plates with elastically suspended screw holes. However, these strategies did not allow for the use of non-locking screws, which are typically used to reduce bone fragments to the plate. This study documents the first in vivo study on the healing of ovine tibia osteotomies stabilized with an advanced active plate (AAP). This AAP allowed plate application using any combination of locking and non-locking screws to support a wide range of plate application techniques. At week 9 post-surgery, tibiae were harvested and tested in torsion to failure to assess the healing strength. The five tibiae stabilized with an AAP regained 54% of their native strength and failed by spiral fracture through a screw hole, which did not involve the healed osteotomy. In comparison, tibiae stabilized with a standard locking plate recovered 17% of their strength and sustained failure through the osteotomy. These results further support the stimulatory effect of controlled motion on fracture healing. As such, the controlled dynamization of locked plating constructs may hold the potential to reduce healing complications and may shorten the time to return to function. Integrating controlled dynamization into fracture plates that support a standard fixation technique may facilitate the clinical adoption of dynamic plating.

## 1. Introduction

The desire to dynamize plates for the promotion of natural bone healing by callus formation predates the 1958 founding of the Swiss AO (Arbeitsgemeinschaft für Osteosynthesefragen), which introduced the first standardized tool and implant set for fracture plating. As early as in 1949, Longfellow was granted a patent for a sliding plate “to permit axial movement for growth of callous”, reasoning that “holding fragments in fixed positions forestalls natural movement between fragments necessary for growth of callous” [1]. Research over the next 50 years consistently showed that controlled axial dynamization promoted callus formation and improved the speed and strength of fracture healing [2,3,4,5,6,7,8,9]. For example, Goodship and Kenwright demonstrated that 1 mm axial dynamization delivered over three times stronger and two times faster healing compared to rigid fixation [5]. Conversely, deficient fracture motion caused by overly stiff fixation constructs can suppress secondary fracture healing, contributing to delayed union, non-union, osteolysis, and fixation failure [10,11,12,13,14]. Consequently, the dynamic fixation of fractures has gained increasing interest secondary to the identification of delayed fracture healing with more rigid constructs [1,2,4,5,9,15]. Before the advent of locked plating, plates needed to be firmly compressed onto the bone surface to obtain stable fixation and efforts to dynamize plates came at the cost of construct stability. The advent of locked plating enabled novel strategies for dynamization, since locked constructs with fixed-angle locking screws no longer relied on the compression of the plate onto the bone surface for stability. These modern clinical solutions to allow early dynamization typically achieve axial motion by dynamizing either screws or plates.

**Figure 1:**
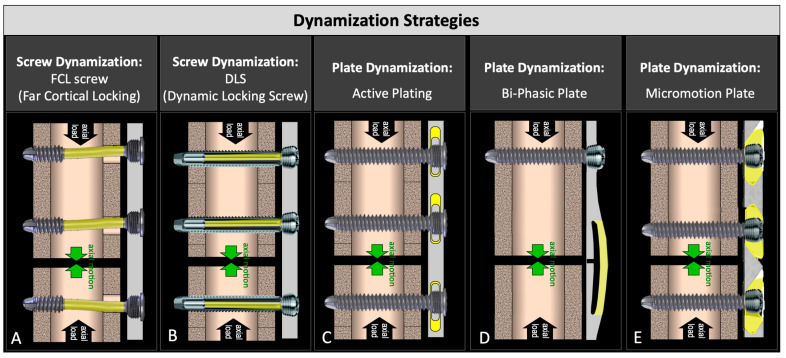
Two screw-based and three plate-based strategies for early and controlled dynamization of fractures: (**A**) far cortical locking; (**B**) dynamic locking screw; (**C**) active plate; (**D**) bi-phasic plate; and (**E**) axial micromotion locked plate (AMLP).

Dynamization with screws was first introduced in the form of far cortical locking (FCL) screws (Figure 1A) [2]. They lock into the plate and the far cortex but retain a motion envelope at the near cortex to enable controlled axial dynamization through the flexion of the screw shaft. FCL screws enabled dynamization without sacrificing construct stability [2]. They delivered symmetric callus bridging and yielded 157% stronger healing compared to standard locked plating in an ovine study [2], and they remain available today for clinical use under the tradename MotionLoc. A similar concept was introduced by Synthes in the form of dynamic locking screws (DLS) that had an elastic pin inside a threaded sleeve (Figure 1B) [16]. DLS screws are no longer available for clinical use since they were recalled by their manufacturer after reports of DLS pin breakage during implant removal. Screw-based dynamization strategies lack scalability to smaller-diameter bones and cannot be combined with compression screws to lag the plate to the bone surface, making widespread adoption difficult.

The dynamization of locking plates was first introduced in the form of active plates in which standard locking screws lock into the threaded hole of elastically suspended sliding elements embedded inside the plate, allowing controlled axial motion (Figure 1C) [17]. Active plates delivered four times stronger fracture healing relative to a locked plate construct in an ovine gap fracture model [17]. In a similar study comparing the fixation of simple fractures with compression plate constructs, active plate fixation resulted in 147% greater healing strength than the compression plate constructs at 9 weeks [18]. A clinical study of 11 humerus fractures stabilized using active plates resulted in early callus formation with excellent clinical outcomes and an early return to function [19]. Recently, a biphasic plate was developed that spans a fracture zone with two beams, whereby the inner beam closer to the bone surface has a transverse slot (Figure 1D) [20]. This enables the elastic flexion of the outer beam until the slot in the inner beam closes and the stiffness increases, resulting in a biphasic stiffness profile. An ovine study using an early version of this biphasic plate showed 45% stronger healing relative to the control plate with a similar geometry but without the transverse slot [21]. An axial micromotion locked plate (AMLP) was developed that utilized a threaded gliding wedge at each screw hole, allowing up to 0.6 mm of controlled axial motion (Figure 1E) [22]. An ovine study comparing the AMLP to a standard locking plate showed that the AMLP plate provided up to 53% stronger healing when used to stabilize a 3 mm gap osteotomy [22]. While all of these plate-based dynamization strategies have demonstrated superior healing compared to standard locking plates, their clinical utility remains limited. Specifically, active plates and AMLP plates do not allow the use of standard compression screws required for a standard lag-and-lock technique, since compression screws would arrest the motion between the plate and bone surface required for dynamization. The biphasic plate design requires the fracture location to coincide with the location of the double beam bridge. As such, the design of dynamic plates must be advanced to deliver controlled axial dynamization while retaining clinical utility. 

Controversy still exists regarding the timing of motion [23]. Motion can be introduced early, allowing controlled motion for callus stimulation immediately after surgery, or late, wherein the fixation construct is initially rigid and becomes dynamic over time. Late dynamization has traditionally been used in the treatment of delayed unions of fractures stabilized with intramedullary nails by removing the interlocking screw to allow more fracture site motion [24]. Recently, a plate with screw hole collars composed of a degradable polymer was proposed that provides initial rigid fixation until the polymer absorbs for late dynamization [25]. Next to early and late dynamization, the technique of reverse dynamization has been proposed, which allows early fracture site motion with a flexible implant that is converted to a rigid construct once fracture callus formation is confirmed [26,27]. While some controversy on the timing of dynamization remains, there is ample evidence that early dynamization promotes natural fracture healing, while delayed dynamization provides a diminishing return. This was again confirmed in the most recent ovine fracture healing study, finding that a delay in the onset of mechanical stimulation retards callus development, while mechanical stimulation in the early post-op phase yields significantly more and stiffer callus [28].

Given this importance of dynamization for the stimulation of natural bone healing, an advanced active plate was recently designed that delivers controlled interfragmentary dynamization while also allowing the use of non-locking and locking screws to accommodate a wide range of plate application techniques. This study employed the established ovine fracture healing model to evaluate the fracture healing and fixation durability provided by this advanced active plating design. Specifically, we hypothesized that advanced active plates would promote circumferential callus formation and bridging by controlled axial dynamization, while allowing the combined use of locking and non-locking screws to support a wide range of plate application techniques.

## 2. Material and Methods

Using an established large animal fracture healing model [29], seven sheep were treated with an advanced active plate for the stabilization of a 3 mm transverse osteotomy gap. This gap model correlates clinically to the bridge plating of a comminuted fracture, in which the entire load is initially transmitted through the plate. Sheep were sacrificed 9 weeks post-surgery, and the treated and contralateral tibia of each sheep were harvested. Callus formation and bridging were evaluated on planar radiographs and by computed tomography (CT). After plate removal, healed tibiae and contralateral tibiae were tested in torsion until failure to determine the strength of the healed tibiae in direct comparison to the native strength of the contralateral tibiae. 

### 2.1. Advanced Active Plating (AAP)

**Figure 2:**
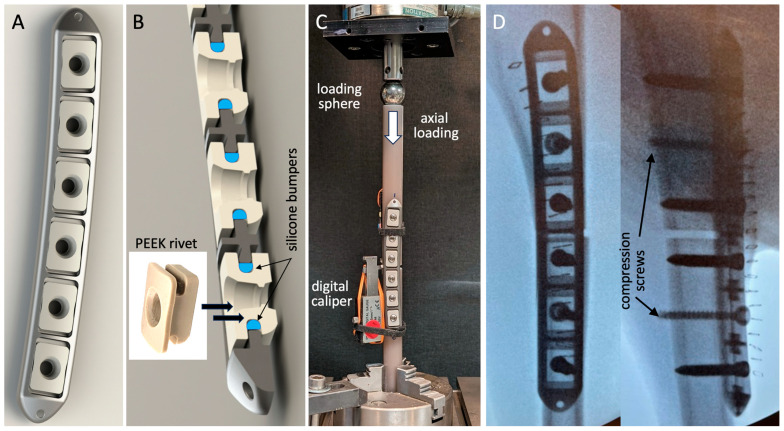
(**A**) Advanced active plate with (**B**) six PEEK anvils that are elastically suspended inside rectangular plate pockets by means of silicone bumpers. (**C**) Stiffness evaluation of the advanced active plating construct. (**D**) Post-operative fluoroscopies, showing plate application with two compression screws and four locking screws to stabilize the 3 mm osteotomy gap.

The advanced active plates had a cross-sectional geometry representative of typical 4.5 mm large-fragment plates (Figure 2A). The six-hole plates with 20 mm hole spacing were 137 mm long, 17 mm wide, and 6 mm thick; were composed of Ti6Al4V titanium alloy; and were type II anodized to improve their corrosion and abrasion resistance. For controlled dynamization, screw holes were located in oblong anvils composed of polyether-ether-ketone (PEEK). These PEEK anvils had elastomeric bumpers composed of implant-grade silicone, which suspend them in a pre-loaded central position in rectangular plate pockets (Figure 2B), enabling the controlled axial motion of the anvils along the longitudinal plate axis. Each PEEK anvil had one central screw hole that accommodated variable-angle 5 mm locking screws and 4.5 mm non-locking compression screws. Since the anvil protruded 1 mm below the bone-facing surface of the plate, compression screws could be used to lag the plate to the bone while retaining controlled dynamization due to the elastic connection between the anvil and the plate by means of the elastomeric bumpers. This AAP design enabled up to 3 mm axial motion across the osteotomy gap while providing stable fixation in response to bending and torsional loading.

The stiffness of the AAP constructs was characterized by the bench testing of three plates, applied to bridge 3 mm gap osteotomies created in cylindrical diaphysis surrogates of 20 mm diameter (3403-23, Sawbones USA, Vashon, WA, USA). Axial compression was applied through a sphere proximally to permit physiological bending under axial loading [16,22,30]. The constructs were stepwise loaded in 50 N increments up to 500 N compression. The resulting motion at the medial and lateral aspects of the osteotomy was measured with digital calipers for the calculation of the construct stiffness, extracted as the slope of the load curve between 200 and 500 N compression (Figure 2C).

### 2.2. Animal Model

The ovine tibia osteotomy model was employed as it represents the most prevalent large animal model for the evaluation of fracture healing [29,30]. The study protocol was approved by the Institutional Animal Care and Use Committee of the Legacy Research Institute. Seven skeletally mature Polypay sheep (3.3 years ± 0.3 years old, 70 ± 13 kg weight) were enrolled. Under general anesthesia, an approximately 8 cm long medial incision was created over the tibia of the right hind leg. To control the osteotomy gap size, six screw holes were drilled in the intact tibia with a custom drill template. A transverse osteotomy was performed with a 0.6 mm thick saw blade under constant irrigation. Osteotomies were stabilized with plates applied to the medial tibial shaft in a periosteum-sparing biological fixation technique by a board-certified orthopedic surgeon to preserve periosteal perfusion [31]. Plates were applied in a standard lag-and-lock technique, whereby each diaphyseal segment was first lagged to the plate with a 4.5 mm compression screw, followed by two additional 5.0 mm locking screws for fixed-angle stabilization (Figure 2D). The resulting osteotomy gap had a controlled width of 3 mm, formed by the distance between the central screw holes in the plates, which was 2.4 mm greater than in the drill template, and by the osteotomy cut of 0.6 mm. For medication, the standard protocol of the ovine fracture model was followed [7,22]. Prophylactic antibiosis with benzylpenicillin and gentamicin and analgesia with carprofen (4 mg/kg BW) and buprenorphine were initiated pre-operatively and were continued for 4 days postoperatively. The surgical limb was protected by a cast applied over a soft padding layer, and the sheep were allowed to ambulate immediately after surgery. As a routine prophylactic measure to avoid the tibial fracture of the operated limb in this ovine model [30,32,33,34], the sheep remained in a protective harness for 4–6 weeks post-surgery. This loosely applied harness allowed full load bearing while standing and walking [34]. The harness was mounted to a roller track, allowing the sheep to ambulate freely throughout their stall. However, the protective harness suspended the animals during sleep to prevent peak loads during sudden bolting from a lying position. Nine weeks after surgery, the sheep were sacrificed for the quantitative assessment of fracture healing. 

### 2.3. Radiographic Assessment of Fracture Healing

Planar antero-posterior radiographs were obtained at week 9 after tibial harvest and the removal of the soft tissue envelope to document callus formation and bridging at the lateral cortex opposite the plate. After plate removal, computed tomography (CT) scans were obtained to render longitudinal cross-sections of the tibial diaphysis depicting callus formation and bridging at the anterior and posterior tibial aspects. Finally, three-dimensional renderings of the CT scans were obtained to reconstruct the callus formation at the medial aspect of the tibia adjacent to the plate.

### 2.4. Mechanical Testing

The proximal and distal ends of the tibiae were cemented in mounting fixtures that were separated by 170 mm and aligned with the tibial shaft axis. To ensure the unconstrained torsion of the tibial shaft in a material test system (Instron 8874, Norwood, MA, USA), the distal fixture was mounted on an x–y table that enabled the translation but prevented the rotation of the distal fixture around the diaphyseal axis. After implant removal, rotation was applied proximally at 10°/minute until specimen failure in torsion. Failure was defined as the instant at which the rotational displacement caused a decrease in the torsional moment due to specimen fracture or shearing at the osteotomy. The strength of the healed tibiae was quantified by their energy to failure, calculated by integrating the area under the torsion versus rotation curve up to the peak torque at which failure occurred [2,17]. The strength of the healed tibiae was furthermore normalized and expressed as a percentage of the native strength of the contralateral tibiae. 

## 3. Results

### 3.1. Construct Stiffness

Bench-top tests demonstrated axial stiffness of the AAP construct of 826 ± 172 N/mm. The application of 500 N axial loading induced 0.7 ± 0.1 mm and 0.6 ± 0.1 mm interfragmentary motion at the lateral cortex opposite the plate and medial cortex adjacent to the plate, respectively. At this load level, the interfragmentary motion approached the 1 mm threshold required for the stimulation of callus formation [3,5,35].

### 3.2. Healing Assessment

**Figure 3:**
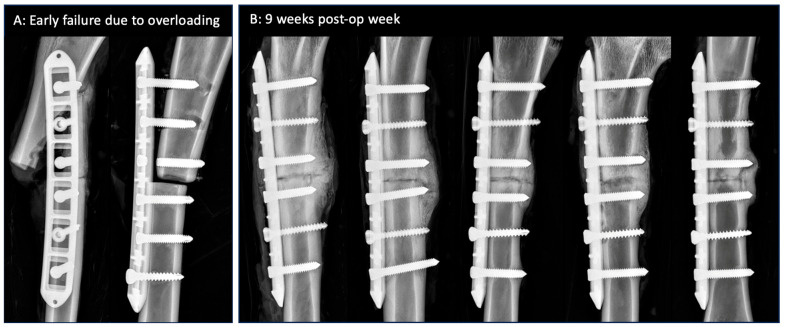
(**A**) Early fixation failure in two sheep due to overloading. Rectangular pockets in the plate are clearly visible since the PEEK anvils are radiolucent. (**B**) Week 9 radiographs of the remaining five tibiae with consistent bridging callus at the lateral cortex opposite the plate.

Five of the seven sheep had an uneventful recovery during the 9 week fracture healing period. Two sheep sustained early fixation failure due to sudden overloading at weeks 1 and 2 post-surgery due to bolting or jumping (Figure 3A). Failure modes included periprosthetic fracture, screw pull-out, and screw breakage. Both sheep were euthanized, and the fixation hardware was retrieved. The AAP plates withstood these traumatic overloading events without bending, the dislodging of anvils, or the loosening of the threaded screw heads from the PEEK anvils.

Antero-posterior radiographs at week 9 demonstrated consistent bridging callus at the lateral cortex opposite the plate (Figure 3B). The plates remained well reduced to the medial surface of the tibial diaphysis. 

**Figure 4:**
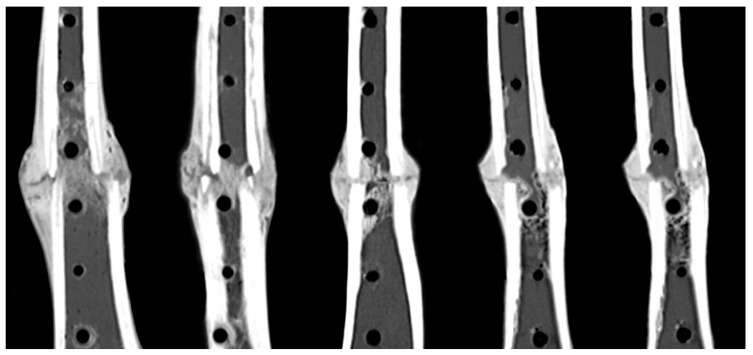
Consistent formation of periosteal callus formation and bridging at the anterior and posterior aspects at week 9 post-op.

Reconstructions of the longitudinal cross-sections of the diaphysis from CT scans demonstrated consistent periosteal callus formation at the anterior and posterior tibial aspects in all five sheep (Figure 4). The smooth periphery of the periosteal callus is indicative of the remodeling phase during the natural bone healing cascade.

**Figure 5:**
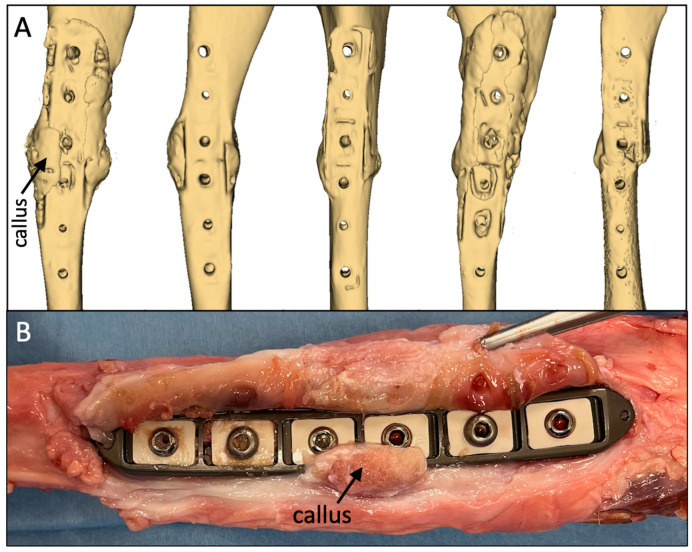
(**A**) Consistent formation of periosteal callus on the medial aspect adjacent to the plate. (**B**) In two out of the five specimens, the medial callus extended partially onto the plate surface but did not adhere to the plate surface.

Callus formation along the plate sides in all five tibiae was evident on the CT reconstructions of the medial diaphysis to which the plate was applied (Figure 5A). This indicated that sufficient axial interfragmentary motion was present to stimulate periosteal callus formation circumferentially around the entire diaphysis. In two of the five specimens, the callus formation extended partially over the plate surface (Figure 5B). In both of these specimens, the periosteal callus did not adhere to the plate surface. The axial motion between the plate and the periosteal surface induced a thin tissue sheath that enveloped each plate. After screw removal, all five plates could be retrieved manually without the need for instruments. The macroscopic evaluation of the tissue sheath surrounding the plate demonstrated no signs of adverse local tissue reactions to the implant materials or wear debris. 

### 3.3. Mechanical Strength

**Figure 6:**
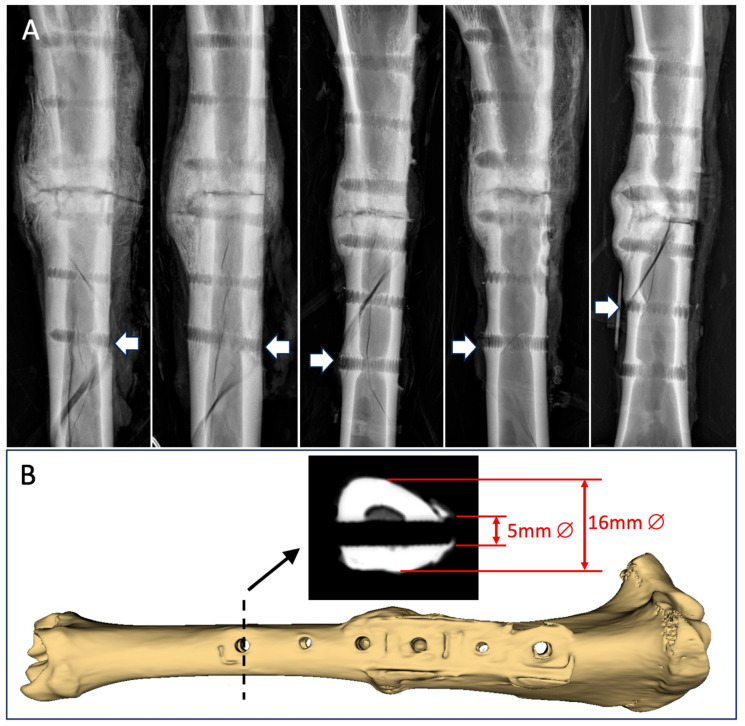
(**A**) All 5 specimens failed by spiral fracture involving a distal screw hole, as depicted by the white arrows. (**B**) The 5 mm diameter screw hole comprised approximately 30% of the diaphyseal diameter.

In the torsion testing for the strength assessment, all five specimens failed by spiral fracture through a distal screw hole, which considerably weakened the diaphysis (Figure 6A). The spiral fractures did not extend to the healed osteotomy in four out of the five specimens, indicating that all but one osteotomy had healed to be stronger than the remaining diaphysis with screw holes. The 5 mm screw hole comprised approximately 30% of the tibial shaft diameter of 16 mm (Figure 6B), which has been predicted to reduce the torsional strength of the diaphysis by approximately 50% [36]. The five specimens had average torsional strength of 42 ± 17 Nm and required energy to failure of 253 ± 141 Nm°. Compared to the intact contralateral tibiae, the osteotomized tibiae regained, on average, 54 ± 19% of their native torsional strength.

## 4. Discussion

The results of this in vivo study confirm that callus stimulation by controlled axial dynamization can be reliably achieved with a plating solution that is clinically practical and flexible. This latest iteration of an advanced active plating design consistently delivered circumferential bridging of a 3 mm osteotomy gap. Circumferential callus formation with the bridging of all four cortical quadrants is a hallmark of dynamic fixation that delivers substantially symmetric gap motion. Conversely, the high stiffness of standard locked plating constructs suppresses axial interfragmentary motion and circumferential callus formation. In a prior study that used the same in vivo model, six tibiae stabilized with a standard locking construct regained, on average, only 17% of their native strength, and all tibiae failed by fracture that involved the osteotomy gap [17]. Similarly, another study employing the same in vivo model found, in three out of six tibiae stabilized with a standard locking plate, that the bridging at the near cortex was deficient, with little or no new bone formation at the osteotomy gap adjacent to the plate [2]. The six tibiae sustained, on average, energy to failure of 111 Nm°, which was 2.5 times lower than the energy to failure of 279 Nm° sustained by tibiae that were treated with an axially dynamic plating construct. Their finding for the axially dynamic group closely correlates with the average energy to failure of 253 Nm° reported in the present study for advanced active plates. 

Advanced active plating allowed, for the first time, the combination of locking and compression screws in axially dynamic plates to aid fracture reduction. This versatility is crucial to facilitate a standard lag-and-lock technique that utilizes a compression screw to lag a bone segment to the plate, followed by locking screws to maximize the fixation strength. Since the anvils of the advanced active plate protrude through the bottom surface of the plate, controlled dynamization is retained despite the compression of the anvil onto the bone surface. A prior active plating generation only supported locked screws and required a staggered screw arrangement [17]. The advanced active plating design also supports the use of uni- or bi-cortical screws, as well as variable-angle screws to optimize the screw trajectories. The design thereby overcomes the limitations of far cortical locking (FCL) screws, which require fixation in the far cortex and do not support variable-angle fixation. Moreover, advanced active plates deliver axial dynamization without requiring a long bridge span, as demonstrated in the present study by the screw placement in close proximity to the osteotomy gap. This contrasts alternative strategies for fracture dynamization that recommend a long or flexible bridge span for stiffness reduction [37]. However, such strategies increase the motion at the cortex opposite the plate but not adjacent to the plate, and they may increase the undesirable shear motion at the fracture site [37]. Furthermore, advanced active plating pertains to the bridge plating of comminuted fractures, as well as to the fixation of fully reduced simple fractures. Anatomically reduced osteotomies in the ovine tibia stabilized with an active plating construct recovered, on average, 64% of their native strength by week 9 [18]. They healed to be over twice as strong as compression-plated osteotomies that recovered only 24% of their native strength [18]. The applicability of active plating for the stabilization of simple fractures was furthermore confirmed in a clinical study that successfully employed active plates for the dynamic fixation of 11 humeral shaft fractures, six of which were simple AO/OTA type 12A fractures [19]. Ten of the 11 fractures healed after an average of 11 weeks, as evident by bridging callus and pain-free function. Compared to the advanced active plate of the present study, the clinical study employed an active plate with a staggered screw pattern that did not allow the use of non-locking compression screws or variable-angle screw placement. Finally, advanced active plating designs may also pertain to periarticular plates, whereby elastically suspended anvils are only employed on the diaphyseal plate segment. By permitting a standard surgical technique, flexibility in screw selection, and a range of potential indications, the advanced active plating design might provide a crucial advancement that combines ease of use, reliable fixation, and controlled dynamization for the promotion of natural bone healing. 

This in vivo model represented a “worst-case” loading scenario whereby the plate endures full load transfer across the osteotomy gap, similar to a comminuted fracture scenario, and unrestricted load bearing immediately post-surgery. Two sheep experienced a periprosthetic fracture from overloading due to sudden bolting, despite the use of a protective harness. This was likely caused by the height-adjustable harness being positioned too close to the ground. To prevent this complication in future studies, the harness should be adjusted to remain in close proximity to the abdomen of the sheep when standing. Under the demanding loading conditions of this in vivo model, the advanced active plates exhibited no fatigue, fracture, wear debris, mechanical failure or other unforeseen complications, supporting the clinical feasibility of their design and materials. The plates even sustained the traumatic overloading events in two sheep that caused a periprosthetic fracture, screw pull-out, and screw breakage. This might be attributed to the efficient lattice structure of the plate, which allows for rectangular pockets while retaining high bending and torsional strength. Moreover, the use of PEEK material for the anvils prevented wear debris, provided a strong interface for the threaded screw heads, and allowed screw removal without the cold welding of the screw head in the screw hole. The elastomeric suspension was composed of long-term implantable silicone, which has been used for over 40 years for finger joint replacements [38] and continues to serve in a range of dental [39], spinal [40], and arthroplasty [38,41] implants to provide elastic fixation, motion, and impact damping. Its use for osteosynthesis implants has recently been cleared by the United States Food and Drug Administration. Given the established clinical history of silicone elastomers and PEEK, these material choices constitute a novel yet conservative strategy to integrate controlled dynamization into an axially dynamic plate design. 

This study had several limitations. It was limited to a 9 week endpoint, consistent with similar studies employing the ovine fracture healing model [2,8,17,18]. While this was a relatively short follow-up period from a clinical perspective, the tibiae had conclusively developed unions, as demonstrated by the mechanical testing after sacrifice. To translate the advanced active plating technology into clinical practice, a prospective clinical study with long-term follow-up will be required, documenting radiographic healing, potential late complications, and patient-reported functional outcomes. This study did not have a control group of standard locking plates. Several studies have already tested standard locking plates in this ovine 3 mm tibial osteotomy model, with highly consistent results, demonstrating that rigid fixation leads to inconsistent and asymmetrical callus formation and deficient healing [2,17]. Moreover, the present study was designed to evaluate the durability of advanced active plates in vivo and to document whether they can consistently stimulate circumferential callus formation and bridging. As such, the rationale for a control group was insufficient to justify the enrollment of additional sheep. While *n* = 7 sheep were enrolled, the results were limited to a sample size of *n* = 5 due to complications in two sheep. The standard sample size of the ovine tibia fracture healing model is *n* = 6 [2,5,8,17,18]. Since this was a descriptive study, focused on evaluating the durability of the fixation hardware and the documentation of fracture healing, the enrollment of additional sheep was not deemed justifiable or necessary. Furthermore, the inclusion of groups representing other dynamization strategies (FCL, active plating) would have allowed for a direct comparison between technologies. However, since these technologies have already been studied in the same ovine model, the animal burden and financial cost for such a large study seemed not to be justified. The results are specific to the ovine 3 mm tibial osteotomy model and therefore require careful interpretation before extrapolation to clinical practice. The ovine model was selected as it represents the most established fracture healing model in large animals [29,30]. Load transmission in the ovine tibia corresponds in magnitude to lower-extremity loading in humans [42]. In this study, load bearing induced the interfragmentary motion needed for mechanical stimulation but the resulting osteotomy motion was not monitored in vivo. For patients that are unable to bear a load, external fixators with actuators for the induction of interfragmentary motion have recently been explored [43]. As a further limitation, the strength assessment was limited to torsion due to the destructive nature of strength tests. Torsion was chosen over bending since the bending strength is highly affected by the rotational orientation of the tibia, while the torsional strength is not. Finally, the findings are specific to fracture fixation in strong bone. Given that osteosynthesis is a race between healing and fixation failure, consistent and circumferential callus healing by axial dynamization may be even more desirable in the presence of osteoporotic bone, where fixation failure is most common. 

## 5. Conclusions

This study demonstrated that the advanced active plate design delivered controlled interfragmentary motion that consistently stimulated natural fracture healing by circumferential callus formation. The combined use of locking and compression screws furthermore supported plate application in a standard lag-and lock approach, thereby improving the clinical utility compared to earlier active plating designs. 

## Data Availability

The raw data supporting the conclusions of this article will be made available by the authors on request.
